# A novel ecological methodology for constructing ethnic-majority life tables in the absence of individual ethnicity information

**DOI:** 10.1136/jech-2014-204210

**Published:** 2015-01-06

**Authors:** Melanie Morris, Laura M Woods, Bernard Rachet

**Affiliations:** Cancer Research UK Cancer Survival Group, Faculty of Epidemiology and Population Health, Department of Non-Communicable Disease Epidemiology, London School of Hygiene & Tropical Medicine, London, UK

**Keywords:** DEMOGRAPHY, ETHNICITY, Health inequalities, STATISTICS, EPIDEMIOLOGY

## Abstract

**Background:**

Deprivation-specific life tables have been in use for some time, but health outcomes are also known to vary by ethnicity over and above deprivation. The mortality experiences of ethnic groups are little studied in the UK, however, because ethnicity is not captured on death certificates.

**Methods:**

Population data for all Output Areas (OAs) in England and Wales were stratified by age-group, sex and ethnic proportion, and matched to the deaths counts in that OA from 2000 to 2002. We modelled the relationship between mortality, age, deprivation and ethnic proportion. We predicted mortality rates for an area that contained the maximum proportion of each ethnic group reported in any area in England and Wales, using a generalised linear model with a Poisson distribution adjusted for deprivation.

**Results:**

After adjustment, Asian and White life expectancies between 1 and 80 years were very similar. Black men and women had lower life expectancies: men by 4 years and women by around 1.5 years. The Asian population had the lowest mortality of all groups over age 45 in women and over 50 in men, whereas the Black population had the highest rates throughout, except in girls under 15.

**Conclusions:**

We adopted a novel ecological method of constructing ethnic-majority life tables, adjusted for deprivation. There is still diversity within these three broad ethnic groups, but our data show important residual differences in mortality for Black men and women. These ethnic life tables can be used to inform public health planning and correctly account for background mortality in ethnic subgroups of the population.

## Introduction

Life tables are a demographic tool used to examine mortality by age and sex. They are the means by which life expectancy at birth is estimated for a given population. It is of public health interest to use life tables to produce accurate estimates of mortality for subpopulations since mortality varies sociodemographically, as we have shown previously^1^ with shorter life expectancy being a feature of neighbourhoods with higher levels of economic deprivation.^2–5^

Health outcomes are also known to vary by ethnicity,^2^ ^6^ ^7^ which is likely to be due in part to lower socioeconomic status being more common among some ethnic minority groups.^2^ ^8^ ^9^ However, some outcomes are worse than would be expected even after taking deprivation into account.^3^ ^5^ ^8^

Improving the health and access to healthcare of ethnic minority groups has long been a goal of government policy to help to reduce overall inequalities in health and mortality,^10^ ^11^ but relatively little has been published on the impact of ethnicity on mortality. In countries where ethnicity is recorded on death certificates it is possible to produce life tables specific to ethnic subpopulations. For example, the US routinely reports large discrepancies in mortality rates by ethnicity,^12–15^ and in New Zealand, ethnic-specific life tables have highlighted the gap between Maori and non-Maori life expectancy.^16^ ^17^ In the UK, however, this individual-level approach is not possible because ethnicity is not recorded on death certificates, despite long-standing calls for its introduction.^10^

In order to assess the impact of ethnicity on health, therefore, studies have attempted various methods to measure ethnicity ecologically and link it to health outcomes, with findings generally highlighting minority ethnic disadvantage.^18–20^ In the absence of a reliable method of assigning ethnicity for each individual person at death, few in the UK have investigated the impact of ethnicity on mortality itself.^6^ ^8^ ^21^

We have used an ecological approach to estimate ethnic-specific life tables for the whole of England and Wales in order to establish a more accurate estimation of the mortality experience of broad ethnic groups. We report the results of these ethnic-majority life tables, discuss the mortality patterns observed and their potential use.

## Methods

To calculate mortality rates, counts of deaths (numerators) and of persons (denominators) are needed by age and sex. To obtain ethnic-specific mortality rates, these counts need to be further stratified by ethnicity. In England and Wales information about ethnic group is not available for individuals who die, but population counts by ethnicity are known for small geographical areas.

### Mortality data

Counts of deaths occurring in England and Wales were provided by the Office for National Statistics (ONS) for the 3 years around the 2001 census (2000, 2001 2002) by Output Area (OA, n=175 434). These are the smallest geographical areas for which data are provided in the Census in 2001. Each OA covers an average population of 300–400 people (although there is a range from a minimum of just under 100 up to around a maximum 4000), so enabling use of the most fine-grained data available. Their boundaries are fixed by ONS so that they are “as socially homogenous as possible based on tenure of household and dwelling type.”^22^ Mortality data were supplied in single-year of age and later grouped to reflect the structure of the population data.

### Population and ethnicity data

Counts of persons by age, sex and ethnicity were provided by ONS from Census 2001 for each OA in England and Wales. Using these data the proportion of persons identifying themselves as White (British, Irish, White other), Asian (Indian, Pakistani, Bangladeshi, Asian other) or Black (Caribbean, African, Black other) in each OA was calculated. The distribution of the Black and Asian populations across England and Wales was very much right skewed, with a large proportion of OAs not containing any of these ethnicities (48.5% of OAs for Asian groups, 62.1% for Black groups). The largest proportion of Black people in any OA was 73%, 98% for Asian and 100% for White. There were not sufficient numbers of persons by OA to generate life tables for persons of Chinese or mixed ethnic origin.

The total proportion of persons in each ethnic group in each OA was rounded to the nearest 1%. However, within each OA we retained the more detailed age band-specific and sex-specific proportions of each ethnicity. The one OA with 98% Asian population is shown as an illustration of how the proportion of each age band might vary within the overall proportion of that ethnic group in that OA (table 1).

**Table 1:** Majority-Asian output area (OA; with the maximum proportion found: 98%), showing the differing proportions within the OA that Asian men make up within in each age band

Age band	Proportion of men in that age band who are Asian (%)
0–4	95
5–15	98
16–29	96
30–49	100
50–64	100
65–74	88
75+	100
All ages	98

### Modelling

We collapsed the OA-specific data to generate separate data sets for Black, White and Asian persons, containing the number of deaths and the population by age group (39 groups: single years of age from 0 to 24 and then 14 5-year age groups from 25–29 to 90+), sex and ethnic proportion (100 groups for each age group). Our final data thus consisted of 23 400 rows of data (39 age groups, 2 sexes, 3 ethnicities by 100 proportions). We used a generalised linear model with a Poisson distribution and restricted regression splines to model, separately for men and women, the deaths adjusted for age and the proportion of persons in each ethnic group. The splines enable us to model the non-linear effect of continuous variables.^23^ ^24^

We fitted models with and without an interaction term between age and the proportion of ethnicity. We further adjusted this model for deprivation, using quintiles of the income domain of the Index of Multiple Deprivation (IMD) 2004 for England and IMD 2005 for Wales (both derived from 2001 data), as a covariate. This income domain score was split into quintiles based on the distribution across England and Wales.

An interaction term between proportion of ethnicity and deprivation was also examined. To determine the significance of a given model we used Akaike Information Criterion (AIC): lower values of this statistic indicate a better model fit. We considered a difference of three or more between one model's AIC and the comparator model as statistically significant.^25^

We used the modelled estimates to predict the age-specific and sex-specific mortality rates for each decile of ethnic proportion up to near the maximum. We have estimated this for specific values (ie, hypothetical areas containing 10%, 20%, … 70%/90%/100%) up to the hypothetical populations whose ethnic proportion equalled the maximum observed proportion for each ethnicity in any OA (73% for Black, 98% for Asian, 100% for White). These rates can thus be interpreted as the predicted mortality rates for a population consisting of, for example, 73% Black people, as near to a ‘completely Black’ population as was found among the OAs in England and Wales in 2001. It is important to understand that these estimates are not for any given OA, but rather for specific values of ethnic proportion. We describe these sets of age-specific and sex-specific mortality rates as ethnic-*majority* life tables: life tables for populations where the majority of persons are Black (73%), or Asian (98%) or White (100%).

We calculated life expectancy between age 1 and age 80 for each ethnicity and each sex. The model and data did not allow us to derive accurate estimates for all ethnicities under age 1 and over age 80 so we adopted a conservative approach and excluded them.

### Comparison of the life tables to existing data

We compared the overall predicted national sex-specific mortality rates from each of our models to those derived from national data.^26^ The ethnic-majority life table for the Asian group was also compared with a South Asian-specific life table that we have previously developed using individually-named mortality data (C. Maringe, personal communication, 2014). These data were derived with the software SANGRA which has good sensitivity (89–96%) and specificity (94–98%) for South Asian names.^27^ Some software packages have attempted to assign Black ethnicity using names as they have for Asian names, but the sensitivity is around 4% and so cannot be reliably used.^28^ ^29^

## Results

Although our *ethnic-majority* life tables are derived for populations containing 73% Black, 98% Asian and 100% White persons, for simplicity we relate our results to Black, Asian and White men and women henceforth.

### Comparisons to existing data

The model's prediction of overall (national) mortality corresponded closely with existing national life tables for both sexes, giving reassurance that the regression model was appropriately specified.

Comparisons to the South Asian life table developed from named mortality data generally showed a good correspondence, with some differences at younger ages. These did not reflect a large discrepancy in absolute number of years in life expectancy: a maximum of around 1.5 years absolute difference, found in the youngest ages, between 1 and 20 years old (data not shown).

### Life table models

**Figure 1:**
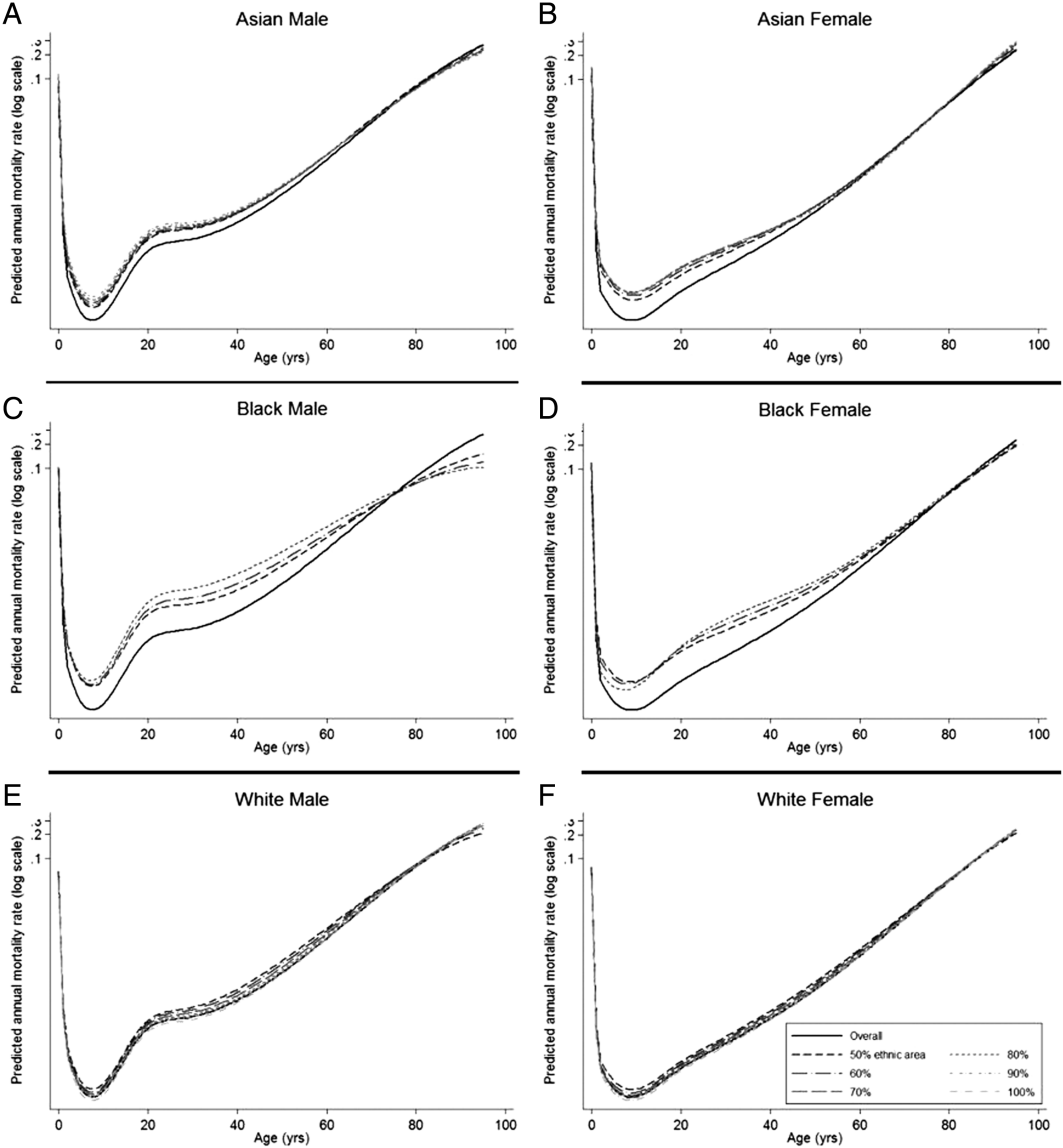
Mortality rates with increasing proportions of ethnicity, from 50% to 90% (Asian; A, male and B, female), 70% (African–American, C+D), 100% (Caucasian, E+F).

The ethnic-majority life table developed from the model for the Black population indicated that each 10% increase in the proportion of Black men (up to 70%) results in higher mortality at all ages up to around 80 years (figure 1). For women, the pattern was more variable with higher proportions of Black ethnicity giving a mortality advantage at younger ages, but a disadvantage over 20 years. In the Asian and White populations, changing the proportion of ethnicity had much smaller impact.

The model including deprivation, without an interaction term with ethnicity, but with an interaction between age and ethnicity, was found to have the best fit to the data. The model was less stable for both sexes at ages over age 80 years, especially for the Black population, as shown by the wider spread of the lines (figure 1). This probably reflects the small numbers in the denominator data for these age groups.

**Figure 2:**
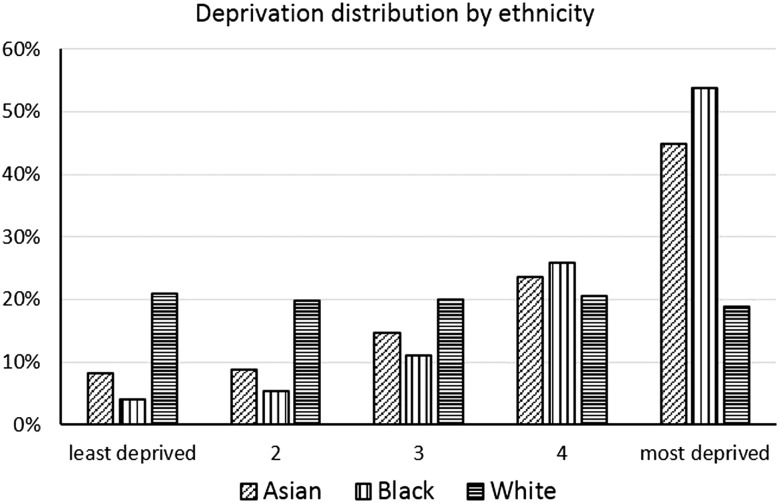
Distribution of deprivation by ethnic group, both sexes combined.

Figure 2 shows the distribution of deprivation quintiles by ethnicity. It is clear that the White group is evenly distributed across the deprivation categories, while the Black and Asian populations are much more concentrated in the more deprived quintiles. The very small proportion of the Black population, for example, in quintiles 1–3, and the strong co-linearity between ethnicity and deprivation may also account for the greater difficulty in fitting models for the Black population. As the model was least stable over 80 years, interpretation of the results (in table 2 and figure 4) was restricted to between 1 and 80 years.

**Table 2:** Probability of surviving between given ages (_n_p_x_), by ethnic group and sex, derived from the deprivation adjusted models

	Ethnic group*					
	Male			Female		
Probability of surviving (_n_p_x_) between:	Black	Asian	White	Black	Asian	White
1–80 (_79_p_1_)	0.394	0.537	0.466	0.593	0.679	0.625
1–20 (_19_p_1_)	0.989	0.991	0.995	0.995	0.994	0.997
20–40 (_20_p_20_)	0.944	0.973	0.980	0.977	0.986	0.990
40–60 (_20_p_40_)	0.845	0.919	0.920	0.925	0.957	0.950
60–80 (_20_p_60_)	0.500	0.605	0.520	0.659	0.723	0.667

*Black refers to an area which is 73% Black, Asian to a 98% Asian area, White to a 100% White area.

**Figure 3:**
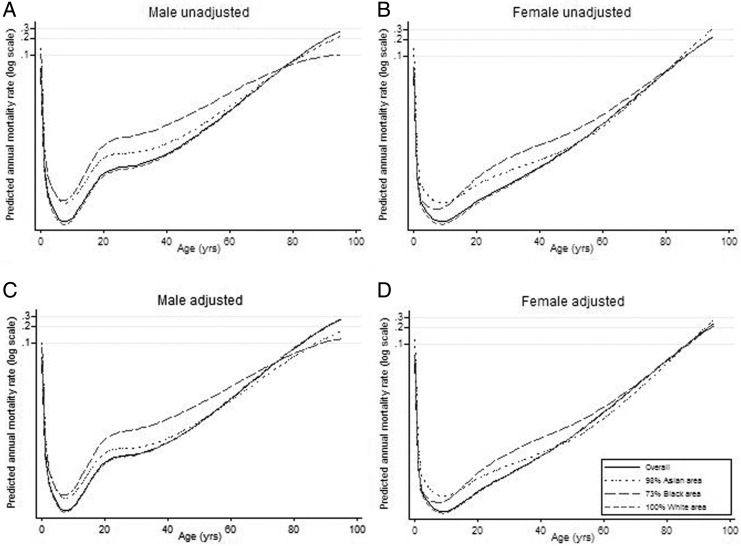
Predicted age-specific mortality rates for populations with the maximum observed proportion of each ethnicity (unadjusted, A and B) and adjusted for deprivation (C and D).

Comparing mortality in men up to the age of 80, White men had the lowest mortality, while Black men experienced the highest mortality rates, whether adjusted for deprivation (figure 3C) or not (figure 3A). The difference between Asian and Black populations was very small up to age 15, after which Black men demonstrated markedly higher mortality until around age 80. In childhood, Asian boys had higher mortality than White boys, but Asian men closed the gap from around age 40 onwards in the unadjusted model. After adjustment for deprivation, predicted mortality rates for Asian men were lower than the White group from age 50.

Before adjustment for deprivation, mortality rates among Asian women were higher than White women up to the age of 50 years. At ages over 50 the rates were very similar (figure 3B). After taking into account deprivation, the mortality rates for Asian and White women became similar around the age of 45 years (figure 3D). Black women had higher mortality than both other groups in adulthood. At ages less than 15 years, mortality among Black girls was lower than for Asian girls.

### Probability of surviving between given ages

Table 2 illustrates the differences in probability of surviving by age group (_n_p_x_) between ethnicities in the deprivation adjusted models, highlighting where some of the differences in mortality arise. While there is a disadvantage for Black men and women in most age groups, it is most marked for men in the age group 40–60. The probability of surviving (_n_p_x_) between 60 and 80 is around 10% higher in Asian men than in Black men, and around 8% higher in Asian men than in White men. A similar but less marked pattern is seen for women.

### Life expectancy

**Figure 4:**
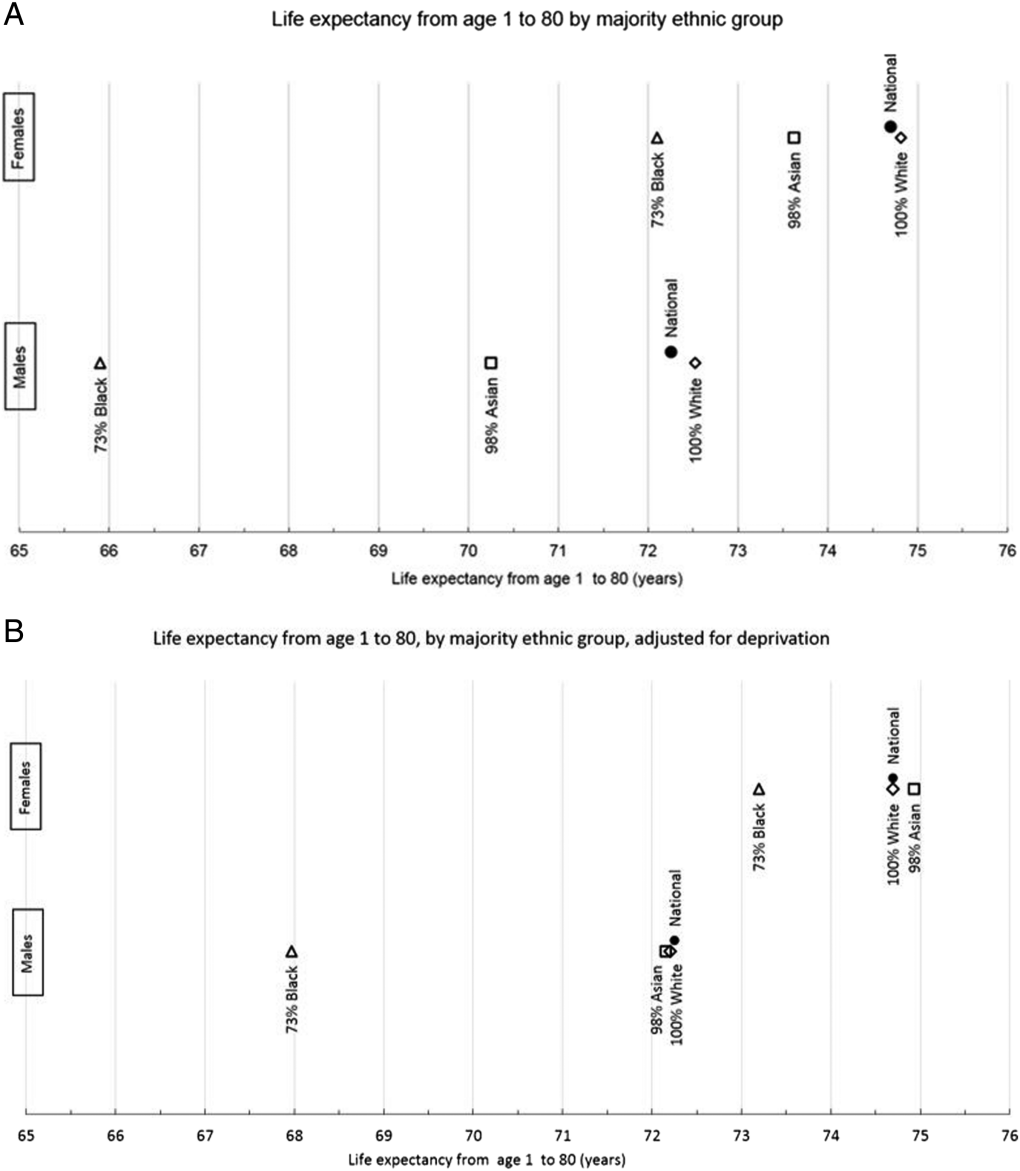
The number of expected years of life from age 1 to 80 for each ethnicity (A) and adjusted by deprivation (B), compared with national figures derived from the model.

Figure 4 shows the number of expected years of life for those aged 1 year up to their 80th birthday (_79_e_1_). Both unadjusted and estimates adjusted for deprivation are displayed. The national figures predicted from the model and estimates for 100% White areas are close because of the very high proportion of White groups across the country.

In the unadjusted model there was a strong advantage in life expectancy for White populations which was particularly evident among men (figure 4A). When the distribution of deprivation is taken into account (figure 2B) the Asian and White groups displayed similar life expectancies, but Black men and women were still at a disadvantage. Black men were 4.2 years behind each of the other groups (_79_e_1_=68.0, 72.2, 72.2). Black women were 1.5 years behind White women (_79_e_1_=73.2 and 74.7 respectively) and 1.7 years behind Asian women (_79_e_1_=74.9; figure 4B).

## Discussion

These data have highlighted stark differences in the mortality experience of ethnic groups in England and Wales. Black men are at a particular disadvantage, displaying a 4-year difference in life expectancy between 1 and 80 compared to Asian and White men after deprivation is taken into account. Black women also have the shortest life expectancy: 1.5 years lower than White women, and 1.75 years lower than Asian women. Asian men and women over age 60 appear to have lower mortality than their White or Black counterparts.

This method, using the proportion of an ethnic group within a very small geography, has been developed due to a lack of individual data on ethnicity in the death register. Scotland introduced the recording of ethnicity on death certificates in 2011,^30^ becoming one of the first countries in the world to do so, however, there is no indication that this will happen in England and Wales in the near future.^31^ Our approach derives from the well-established method for creating deprivation-specific life tables based on ecological data for the small area in which a person lives.^32^ Consequently, it should be remembered that these ecologically-derived life tables do not reflect the mortality experience of a single person living in that ethnic-majority area, but the mortality of a majority Black, Asian or White population. The approach adopted here has been made possible by a new method we have developed of deriving life tables using regression splines.^24^ This allows the modelling of the mortality rates directly from the data using a flexible function for age rather than relying on a model life table approach.^32^ ^33^ The model allows us to use data collated across all OAs to predict the mortality rates that would be experienced by any proportion of ethnic grouping. We have chosen, however, to report predictions at the maximum levels for each group, rather than go beyond reality and report results for putative 100% Black or Asian areas. It should be remembered, though, that the results represent predictions from the model and not the results gleaned from individual OAs directly.

The comparison of our ecological data to name-based individual-level South Asian data provides some support for our results, the similar patterns suggesting the model was working appropriately. There were also some differences but these are likely explained by the variation in methods and the slight difference introduced by including all Asians rather than just South Asians. The differing results also highlight a need for caution. Our decision to group the population into three main ethnicities, by necessity of numbers, will have missed the diversity in health outcomes still to be found within these broad ethnic groups.^34^ Indeed, these broad categories give little information regarding the cultural or faith differences within them. These might have a large impact on healthcare-seeking behaviour, lifestyle choices and support structures.^20^ ^35^ Other studies which used finer categories have suffered from problems with small numbers, but have still highlighted similar patterns to those found here; for example, the mortality disadvantage of Black groups.^6^ ^8^ ^21^ Individual ethnicity data would provide the opportunity to develop life tables that would reflect exactly the mortality experience of each smaller ethnic group. However, by using data from small areas collated by proportion of ethnic group, our results are a closer reflection of the Asian or Black mortality experience than anything else currently available.

Our unadjusted results highlight differences in mortality between ethnic groups, but the impact of socioeconomic status is also notable. Asian populations tend to have lower incomes^2^ and are less likely to be in managerial and professional occupations^9^ and our adjusted results show that deprivation largely explains the Asian groups’ mortality disadvantage. The lower mortality found in our data in the Asian groups could be due to people in poor health leaving to return to their country of origin (the so-called ‘salmon bias’^36^), but it could potentially reflect a real selection effect by which those who have survived through and beyond younger ages display lower risks of death.

In contrast, despite the fact that over three-quarters of the Black population is within the two most deprived quintiles, their mortality difference remains after adjustment. Though this may be due to genetic differences or some unaccounted for lifestyle factors that put them at higher risk of some diseases,^34^ crucial roles played by unmeasured socioeconomic dimensions, including worse living and working conditions,^3^ ^5^ cannot be ruled out.

Our results are consistent with a study using data from the ONS Longitudinal Study which found that almost all the mortality disadvantage in ethnic minority groups was accounted for by differences in socioeconomic status,^8^ except for those of Black Caribbean descent born in the UK. Their study also highlighted the likelihood of a distinction between the mortality experiences of the immigrant versus the UK-born population within the ethnic groups in our data. We were unable to assess this in our data. However, our study has the advantage of using data from the whole of England and Wales, rather than survey data covering only around 1% of the population. We also used the income domain of the IMD measure of deprivation which is more comprehensive than the Carstairs index.^37^ It reflects material deprivation (as opposed to housing, educational or health deprivation) and is a commonly used resource-based measure of socioeconomic position.^32^

Our aim in this study has been to produce life tables that can be used to examine the mortality experience of three broad ethnic groups. While other studies have used other methods successfully to show ethnic difference in health outcomes, often using country of birth as a proxy for ethnicity^8^ ^38–40^ few have attempted to produce life tables themselves. Rees *et al*^6^ have developed ethnic life tables using ethnicity data from local authorities (populations varying from 10 000 s to 100 000 s) with a geographically weighted method.^41^ However, this method seemed to produce over-smoothed results, which may have been a result of the large heterogeneous areas they were using. They rejected this method in favour of another which used Census self-reports of limiting long-term illness as a proxy from which to extrapolate mortality rates at the level of local authorities. There is some debate as to how well this links to mortality, or if it does so in the same way in all ethnic groups.^42^ ^43^ Comparison with our study is also difficult as they have maintained the breakdown of 16 different ethnic groups, and report life expectancies from birth, not adjusted for deprivation. Overall, their results show higher life expectancies throughout for all these groups than our results show for the broader categories after adjustment.

Our focus on life expectancy between 1 and 80 years removes the disproportionate effect that infant mortality has on life expectancy at birth. Others have also advocated this method,^8^ ^44^ especially as there are marked differences between infant mortality rates in different ethnic groups beyond the effect of deprivation.^45^ These differences, however, would be very interesting to examine further if we could access reliable individual death data for all infants by ethnicity.

As the ethnic composition of England and Wales changes we expect the results found here to change and it will be important to examine trends in mortality over time. This analysis forms a benchmark against which future research of this nature can be evaluated.

## Conclusion

Inequalities in health outcomes between deprivation groups are well documented, but there has been less quantification of ethnic differences and this approach gives an opportunity to rectify this. This is the only study that we are aware of which estimates the mortality experience of ethnic minorities across the whole of England and Wales at the level of OA. Our ecological approach uses data at a fine level of detail, applying a novel modelling approach to predict the mortality of populations of whom the majority report to be of a particular ethnic group. The resulting ethnic-majority life tables provide a realistic approximation of the mortality experience of ethnic minorities in the absence of individual mortality data. They can be used to inform public health planning and for other purposes, such as the accurate estimation of net survival from cancer and other important diseases.^16^ ^46^

**What is already known on this subject?**

- Inequalities in health outcomes between deprivation groups are well studied. Mortality inequalities are evident in deprivation-specific life tables. Health outcomes are known to also vary by ethnicity, which is in part to be due to socioeconomic differences. Ethnic groups may have different mortality experiences independent of deprivation. This has not been studied comprehensively because ethnicity is not captured on death certificates.

**What this study adds?**

- Using a novel ecological method involving new modelling techniques, we have developed ethnic-majority life tables which show that Black groups have a mortality disadvantage compared to White and Asian groups, particularly for men. After adjustment for deprivation, the disadvantage observed for Asian groups disappears. These results can help guide resources towards improving the health of certain minority groups. Life tables can also be used to correctly account for the background mortality in ethnic subgroups of the population.
